# APOBEC3G acts as a therapeutic target in mesenchymal gliomas by sensitizing cells to radiation-induced cell death

**DOI:** 10.18632/oncotarget.17348

**Published:** 2017-04-21

**Authors:** Yu Wang, Shaofang Wu, Siyuan Zheng, Shuzhen Wang, Arjun Wali, Ravesanker Ezhilarasan, Erik P. Sulman, Dimpy Koul, W.K. Alfred Yung

**Affiliations:** ^1^ Brain Tumor Center, Departments of Neuro-Oncology, The University of Texas MD Anderson Cancer Center, Houston, Texas, USA; ^2^ Genomic Medicine, The University of Texas MD Anderson Cancer Center, Houston, Texas, USA; ^3^ Radiation Oncology, The University of Texas MD Anderson Cancer Center, Houston, Texas, USA; ^4^ Department of Neurosurgery, Peking Union Medical College Hospital, Chinese Academy of Medical Sciences and Peking Union Medical College, Beijing, China

**Keywords:** mesenchymal gliomas, APOBEC3G, TGFβ signaling, radiation and cell death

## Abstract

Genomic, transcriptional, and proteomic analyses of brain tumors reveal that subtypes differ in their pathway activity, progression, and response to therapy. We performed an expression profiling of Glioma Initiating Cells (GICs) and comparative analysis between different groups of GICs indicates major variations in gene expression. Hierarchical clustering analysis revealed groups of GICs reflecting their heterogeneity, and among some of the genes as major regulators of mesenchymal phenotype, we identified ABOBEC3G as one of the most discriminating genes in mesenchymal group. ABOBEC3G revealed a strong correlation with overall survival in TCGA GBM patient cohorts. APOBEC3G regulates cell invasion and silencing of this gene in GICs inhibits cell invasion and also glioma sphere initiation. APOBEC3G controls invasion through TGFβ/Smad2 pathway by regulating Smad2 target genes Thrombospondin 1, matrix metallopeptidase 2 and TIMP metallopeptidase inhibitor 1. We also show that targeting APOBEC3G can sensitize cancer cells to radiation induced cell death by attenuating activation of the DNA repair pathway. This response is mainly shown by decreased pChk2 expression in knockdown APOBEC3G cells. Taken together, we show that APOBEC3G gene is a mesenchymal enriched gene that controls invasion and knockdown of APOBEC3G sensitizes cells to radiation induced cell death, suggesting that APOBEC3G can be considered for use in stratifying patients with GBM for prognostic considerations.

## INTRODUCTION

Glioblastoma (GBM), the most common primary malignant tumor of the central nervous system [1], is also most aggressive, with a dismal prognosis. The median survival time of patients with GBM is less than 2 years despite standard care, composed of resection, concomitant radiotherapy plus temozolomide, and adjuvant chemotherapy with temozolomide [2]. Few patients survive more than 5 years [3].

Although a great effort has been made to understand the molecular mechanism of GBM, no meaningful survival improvements have been resulted. Recent research has been focused on identifying new targets against GBM [4]. The Cancer Genome Atlas (TCGA) divided GBM into four subtypes: classic, mesenchymal, neural, and proneural [5]. Transcriptional profiling studies by Phillips et al. [6] and Verhaak et al. [5] have revealed molecular subtypes of high-grade gliomas based on the expression of genes characteristic of proneural (PN), neural (N), classical (CLAS) or mesenchymal (MES). A parallel comparison of these two studies revealed particularly strong agreement in the gene signatures associated with the PN and MES subtypes [7]. A number of transcription factors, including C/EBP-β (CCAAT-enhancer-binding protein-β) and STAT3 (signal transducer and activator of transcription 3) and more recently the transcriptional coactivator TAZ (transcriptional coactivator with PDZ-binding motif), have been identified as important regulators of the mesenchymal phenotype in GBM [8, 9].

The APOBEC (apolipoprotein B mRNA editing catalytic polypeptide-like) family of proteins are a group of DNA-editing enzymes that play an important role in the innate immune response to retroviruses and retrotransposons [10]. APOBEC3G is a member of this family that can restrict HIV1 infection by viral strains that lack viral infectivity factor [11, 12]. During reverse transcription in HIV-infected cells, the virion-packaged APOBEC3G deaminates cytidine residues to uridine in minus-strand DNA and causes guanosine-to-adenosine hypermutation in the opposite strand, resulting in inactivation of the viral genome [13–15]. APOBEC3G inhibits elongation of HIV-1 reverse transcripts by inhibiting the translocation of reverse transcriptase along template RNA [16]. In addition, A3G sensitized cells to recognition by NK cells through upregulation of the expression of NKG2D ligands in HIV-infected cells to enhance the immune response and help the defense against pathogens [15]. Apart from contributing to immunity against viral infection, recent studies suggested APOBEC3G also exerts important non-antiviral functions in cancer. APOBEC3G induces oncogenic transformation [17] and promotes liver metastasis and is correlated with poor prognosis in colon carcinoma patients with hepatic metastasis [18, 19]. While APOBEC3A and APOBEC3B are directly linked to cancer through a DNA deaminase-dependent mechanism [20], this is not the case for APOBEC3G as it is retained in cytoplasm [21]. Actually, the mechanism of APOBEC3G in tumors, especially in GBM, is largely unknown.

Transforming growth factor-β (TGFβ) has a key role in tissue homeostasis and cancer, and elevated TGFβ activity has been associated with poor clinical outcome in high-grade glioma [22, 23]. TGFβ can promote epithelial-to-mesenchymal transition in epithelial cancers, leading to enhanced migration and invasion capacities in these cells [24, 25]. It is conceivable that the mesenchymal subtype of GBM has similar mechanisms [26]. TGFβ is highly activated in the mesenchymal subtype of GBMs, but whether APOBEC3G has crosstalk with TGFβ remains to be elucidated. APOBEC3G has been shown to enhance lymphoma cell radioresistance by promoting DNA repair [27]. Since radiation therapy is one of the backbones of the treatment of GBM, whether APOBEC3G can promote radioresistance in GBM is clinically significant. Irradiation (IR) mainly causes cell death through the induction of DNA double-strand breaks (DSBs), which activates the DNA repair pathway. Checkpoint kinase 2 (Chek2, Chk2) is an important signal transducer of cellular responses to DNA damage. Activation of Chk2, especially when it is phosphorylated at threonine 68, can initiate a multistep dynamic process to repair DNA damage [28, 29].

In this study, we screened GICs and TCGA data and found that APOBEC3G was highly expressed in mesenchymal GBM; it was also associated with significantly decreased survival time in GBM patients, suggesting that it is a tumor-promoting factor in GBM. We identified APOBEC3G as a survivor factor of mesenchymal GBM after radiation. We show TGFβ signaling pathway regulated by APOBEC3G that was associated with enhanced tumor invasion in GBM. Targeting APOBEC3G sensitizes GBM cell lines to IR by attenuating activation of the DNA repair pathway.

## RESULTS

### APOBEC3G Is highly expressed in mesenchymal subtype of GICs and GBM cell lines

Transcriptional analyses of brain tumors divide glioblastomas into different subtypes with characteristic pathway activity and response to therapy. Based on the transcriptome array analyses, we found that genes are differentially expressed between Mesenchymal GICs and other non-Mesenchymal GICs. We compared mesenchymal and non-mesenchymal GIC lines (*n* = 7 and *n* = 19, respectively) using the significance analysis of microarrays [30]. Applying a FDR of ≤ 0.01, we identified 89 genes significantly upregulated and 164 genes downregulated in mesenchymal GICs (Supplementary Dataset 1). Among the upregulated genes, APOBEC3G showed strong upregulation in the mesenchymal subtype (fold change = 8.94) (Supplementary Table 1). Of the 89-upregulated genes, 87 were included in the TCGA U133 platform and 83 (95.4%) also showed upregulation (fold change > 1) in the mesenchymal subtype. Among the downregulated genes, 154 were included in TCGA U133A platform, 147 of which (95.4%) showed downregulation. Among the upregulated genes, APOBEC3G also showed strong upregulation in the mesenchymal subtype (fold change = 1.84) (Supplementary Dataset 2, Supplementary Figure 1).

**Figure 1:**
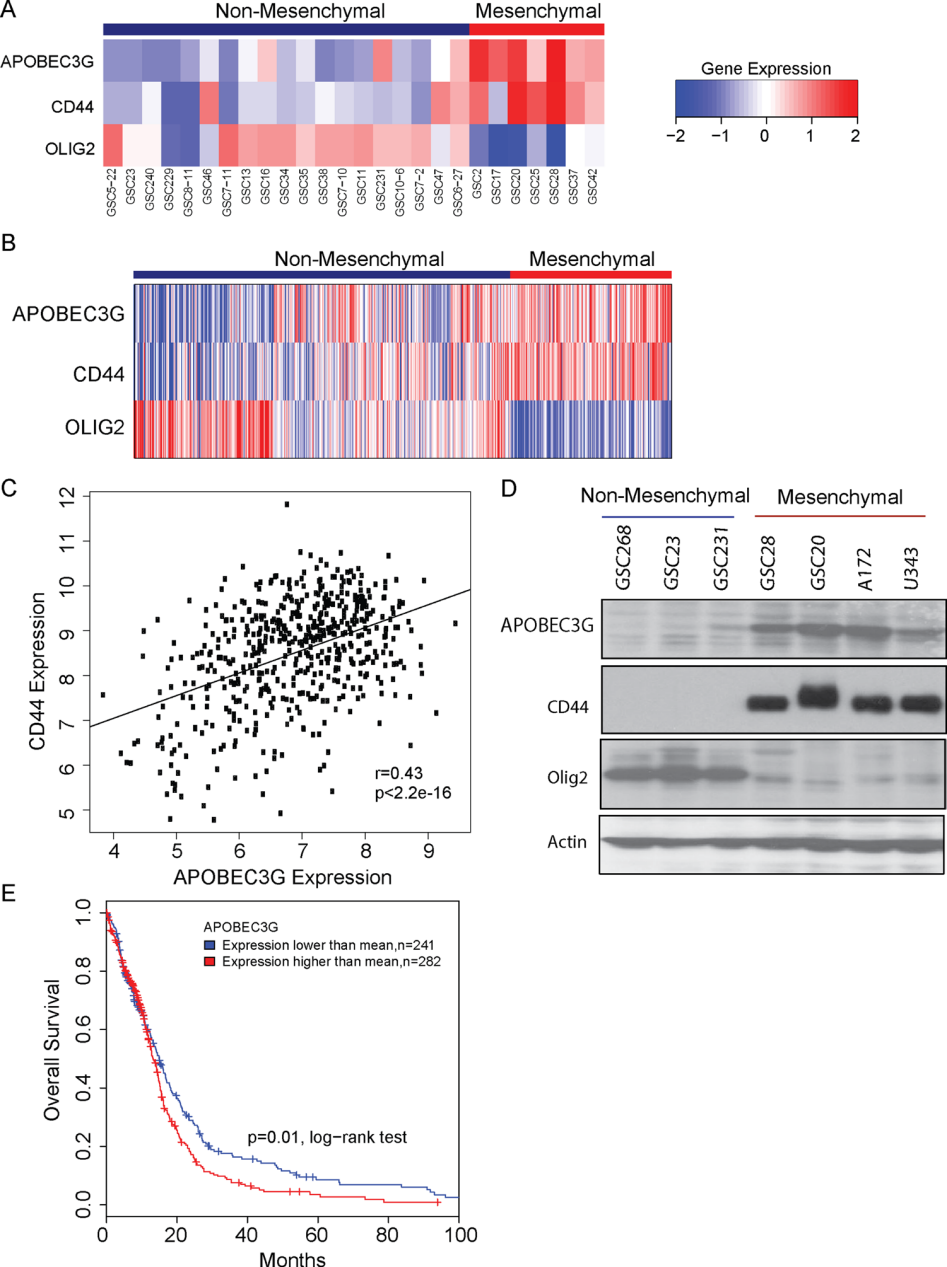
APOBEC3G was highly expressed in mesenchymal subtype of GICs and GBM cell lines (**A**) Gene expression analysis of APOBEC3G in a panel of 26 GICs are shown in the heat map. (**B**) Gene expression analysis of APOBEC3G in TCGA samples are shown in the heat map. CD44 is a marker of the mesenchymal subtype, whereas Olig-2 is the non-mesenchymal marker. (**C**) Expression of APOBEC3G was correlated with CD44 in the TCGA data. (**D**) The expression of APOBEC3G, CD44 and Olig2 in GICs (GSC268, GSC23, GSC231, GSC28, GSC20) and in GBM cell lines (A172, U343) was detected by Western blot analysis. Actin was used as the control. (**E**) TCGA data showed that patients with high expression of APOBEC3G had short survival durations.

Analysis of expression of GICs (Figure 1A) and TCGA data (Figure 1B) revealed that APOBEC3G was enriched in mesenchymal subgroup of GBMs with CD44 as the mesenchymal marker and Olig-2 as a non-mesenchymal marker. We found the expression of APOBEC3G was correlated with CD44 (Spearman's correlation: 0.45) (Figure 1C). To validate the mRNA expression data, we used Western blot analysis to confirm the protein expression of APOBEC3G in several GICs and GBM cell lines. Concordant with the TCGA patient microarray data, APOBEC3G protein was highly expressed in mesenchymal GBM cell lines and GICs but not in non- mesenchymal GICs (Figure 1D). Kaplan-Meier plots and log-rank survival analyses showed that the median overall survival time of the high-APOBEC3G group was markedly shorter than that of the low-APOBEC3G groups, suggesting that APOBEC3G is associated with poor clinical outcomes (*P* < 0.01) (Figure 1E).

### Targeting APOBEC3G attenuates proliferation of mesenchymal GICs and GBM cells

**Figure 2:**
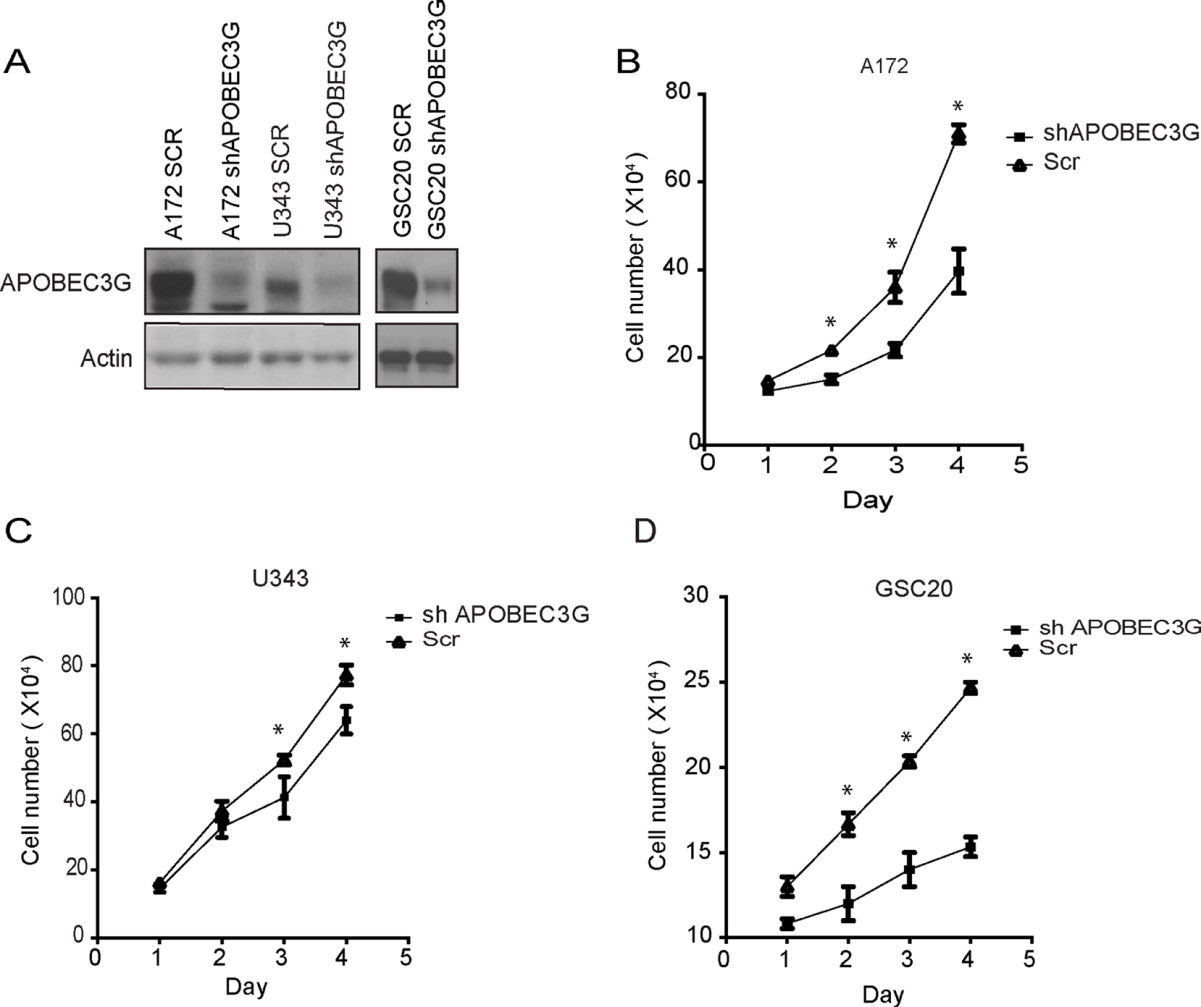
Depletion of APOBEC3G attenuated proliferation of mesenchymal GICs and GBM cells (**A**) APOBEC3G was knocked down by lentivirus shRNAs in A172, U343 and GSC20, and the knockdown effect was confirmed by Western blot. Scramble sequence shRNA (SCR) was used as the control. (**B**–**D**) A172 (B), U343 (C) and GSC20 (D) APOBEC3G knock-down cells as well as SCR cells were seeded in 6-well plates (1 × 10^5^ cells/well), cell numbers were counted and plotted every day for 4 days. Symbol * means *p* < 0.01.

To assess the functional significance of the relative overexpression of APOEC3G in CD44^+^ mesenchymal glioma cells compared with CD44^−^ glioma cells, we depleted APOBEC3G expression using lentivirus expressing shRNA directed against APOBEC3G in A172, U343, and GSC20 cells (Figure 2A). Knock-down of APOBEC3G expression significantly inhibited cell proliferation in A172 (Figure 2B), U343 (Figure 2C), and GSC20 cells (Figure 2D) in comparison to scramble shRNA (SCR) transfected cells.

### APOBEC3G knockdown attenuates invasion of mesenchymal GICs and GBM cells

**Figure 3:**
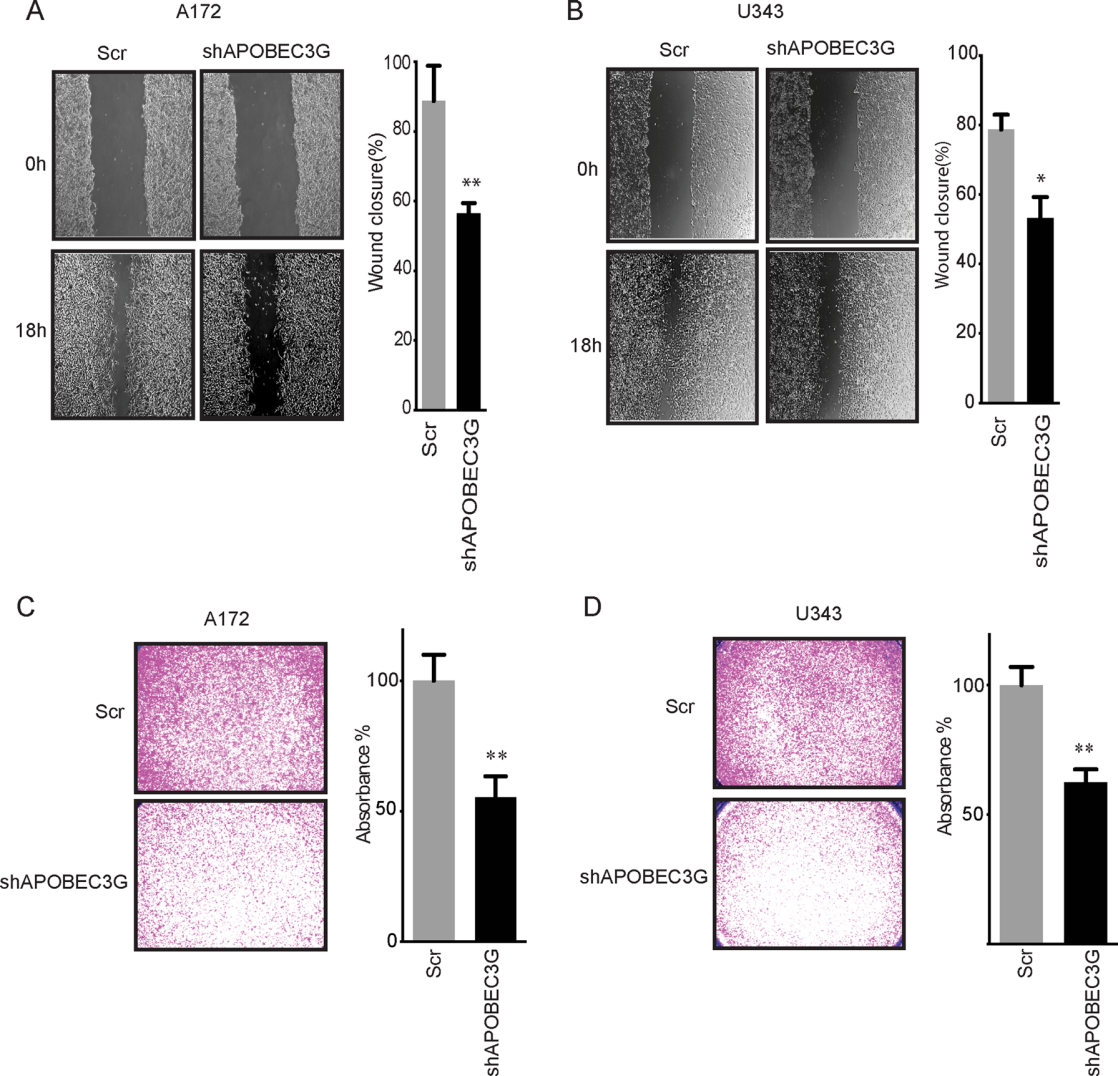
Targeting APOBEC3G impaired migration ability of mesenchymal GBM cell lines (**A**–**B**) Micrographs showing wound healing assay of A172 (A) and U343 (B) APOBEC3G-depleted cells and SCR cells. The width of scratch at 0 h was considered to be 100%. The relative migration distance of cells was demonstrated. (**C**–**D**) Micrographs showing matrigel transwell migration assay in A172 (C) and U343 (D) APOBEC3G-depleted cells and SCR cells. The cells migrated to the lower surface were quantified on the basis of the absorbance measured at 595 nm. The absorbance of transwell of SCR cells was considered to be 100%. Symbols * and ** mean *P* < 0.05 and *p* < 0.01, respectively.

Recent data suggest that APOBEC3G can promote liver metastasis in colorectal cancer [19], but its role in GBM is not clear. To explore the role of APOBEC3G in the migration of GBM cell lines, we performed a wound-healing assay in A172 and U343 cells. Knockdown of APOBEC3G impaired the migration of A172 cells by 36.3% (*P* < 0.01) (Figure 3A) and U343 cells by 32.4% (*P* < 0.05) (Figure 3B) in comparison to scramble control cells after 18 h of observation. We further studied the invasion ability of glioma cells and performed matrigel transwell migration assays to demonstrate that knockdown of APOBEC3G decreased the number of cells that migrated to the other side of transwell by 50.8% in A172 cells (*P* < 0.01) (Figure 3C) and 49.1% in U343 cells (*P* < 0.01) (Figure 3D). These results clearly show that targeting APOBEC3G attenuated the migration and invasive capabilities of GBM.

### Targeting APOBEC3G attenuates TGFβ signaling pathway

**Figure 4:**
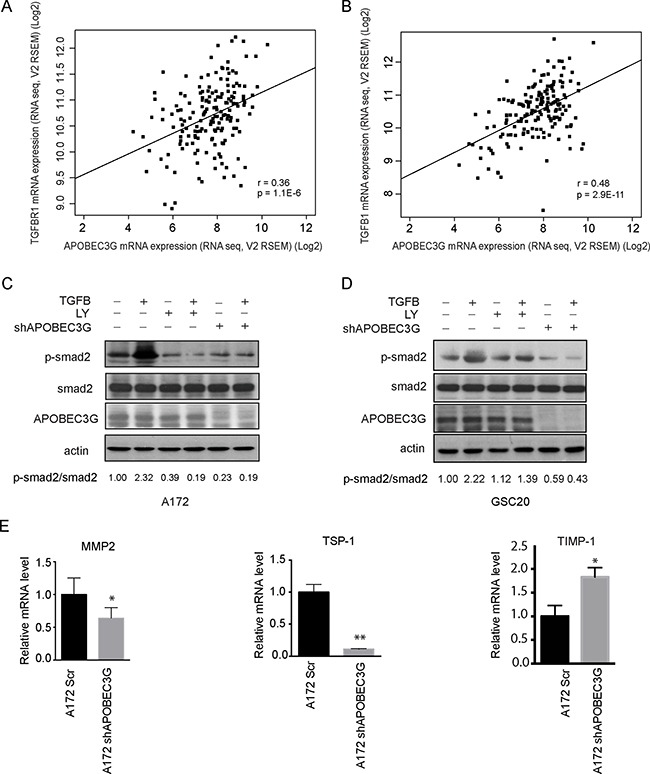
Depletion of APOBEC3G attenuated the TGFβ signaling pathway (**A**–**B**) TCGA data showed that expression of TGFβR1 (A) and TGFβ1(B) was correlated with APOBEC3G. (**C**–**D**) Immunoblotting analysis showed that shRNA targeting APOBEC3G reduced the expression of phosphorylated smad2. A172 cells (C) and GSC20 (D) transfected with scramble or APOBEC3G shRNA, treated with 2.5 ng/ml TGFβ1, 10 mM LY2157299 (TGFβR1 inhibitor), or combination for 24 h, and cell lysis was analyzed by western blot with specific antibodies against phosphorylated smad2, total smad2 and APOBEC3G. The expression of p-smad2 and total smad2 was quantified by Image-J and their ratio was shown in the bottom. (**E**) The expression of smad2 downstream target genes MMP2, TSP1 and TIMP-1 were analyzed in A172 cells transfected with scramble shRNA (A172SCR) or A712 APOBEC3G knock-down cells (A172 shAPOBEC3G) by quantitative-PCR. Symbols * and ** mean *P* < 0.05 and *p* < 0.01, respectively.

To delineate the molecular mechanisms through which APOBEC3G regulates CD44^+^ glioma cell proliferation and migration, we investigated intracellular signaling pathways in APOBEC3G knockdown cells. TGFβ expression in malignant brain tumors was found to render the tumor cells survival advantage by enhancing cell growth, migration, invasion, angiogenesis, and immune suppression and stem cell properties. We analyzed the C-Bio database and data analysis showed expression of APOBEC3G correlated with the expression of TGFβR1 (Spearman's correlation: 0.36) (Figure 4A) and TGFβ1 (Spearman's correlation: 0.48) (Figure 4B). These preliminary data prompted us to further investigate the mechanism of APOBEC3G focusing on TGFβ signaling pathway.

Canonically, TGFβ1 binds and activates TGFβR, which phosphorylates Smad2, the key node of TGFβ signaling pathway. In A172 cells and GSC20 cells, TGFβ treatment induced Smad2 phosphorylation (Figure 4C and 4D), and as expected, the TGFβ-induced Smad2 phosphorylation was abrogated by TGFβR1 inhibitor LY2157299. Interestingly, knockdown of APOBEC3G by shRNA also markedly blocked TGFβ-induced Smad2 phosphorylation (Figure 4C and 4D), suggesting APOBEC3G is involved in TGFβ signaling pathway.

To further understand the role of APOBEC3G in Smad2 deactivation in gliomas, we determined its role in mediating the expression of the Smad2-targeted genes Thrombospondin 1 (TSP-1), matrix metallopeptidase 2 (MMP2) and TIMP metallopeptidase inhibitor 1 (TIMP-1). RT-PCRs were performed to compare the expression levels of Smad2-targeted genes between scramble and APOBEC3G shRNA transfected A172 cells. The results show that the expression of MMP2 and TSP-1 which play important role in cell migration, was decreased in shAPOBEC3G cells compared to scramble controls (Figure 4E, left and middle panels); in contrast, expression of TIMP-1, an natural inhibitor of the matrix metalloproteinases was upregulated by APOBEC3G depletion (Figure 4E, right panel). Taken together, these results suggested that APOBEC3G interferes with TGFβ signaling by affecting Smad2 phosphorylation and Smad2 downstream target gene expression, which may account for APOBEC3G mediated proliferation and migration in mesenchymal GICs and GBM Cells.

### APOBEC3G knockdown sensitizes mesenchymal GICs and GBM cells to IR

**Figure 5:**
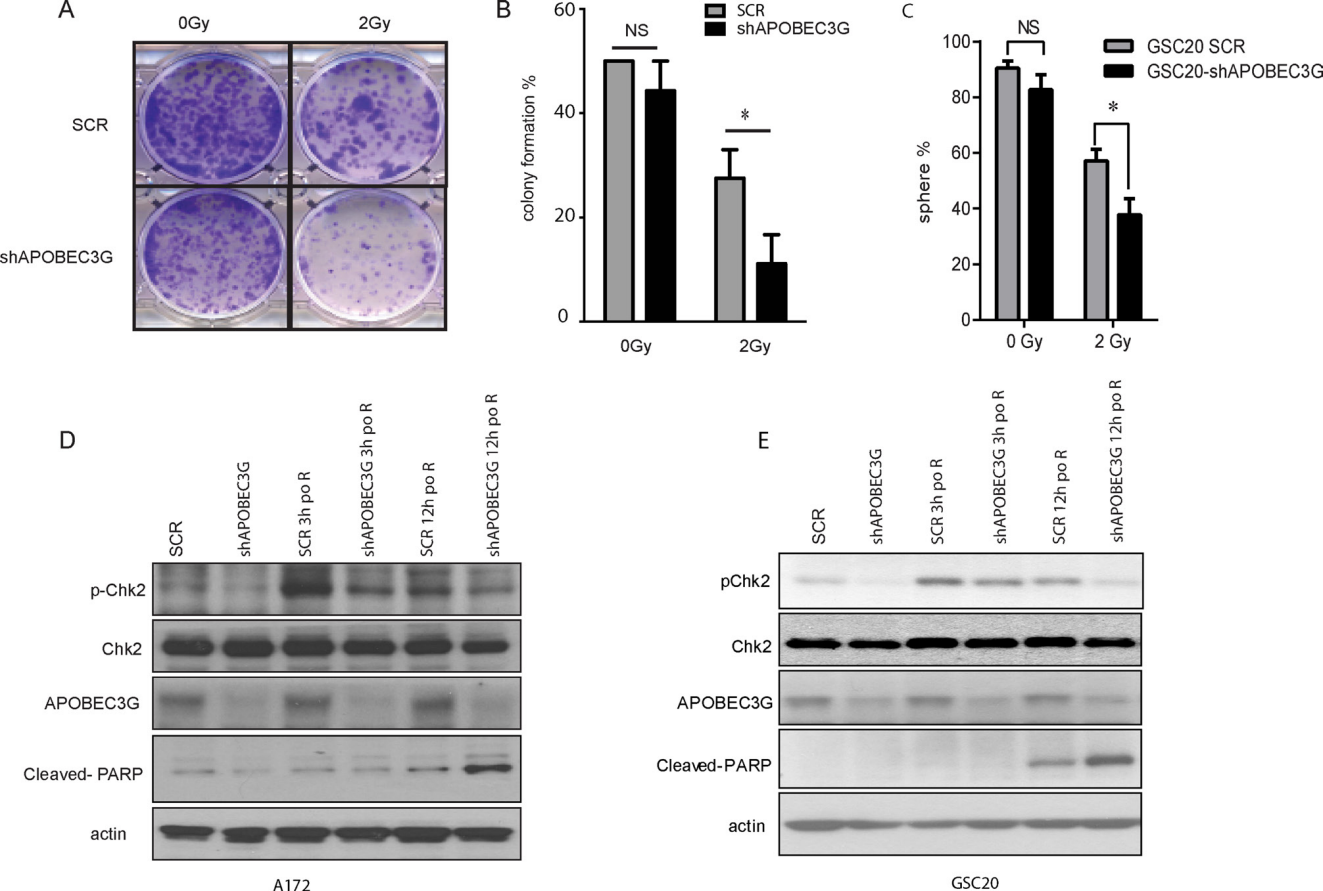
Inhibition of APOBEC3G increased the radiosensitivity of GBM cells and mesenchymal GICs (**A–B**) Colony formation assay showed that APOBEC3G knockdown reduced the colony formation of A172 cells after IR (2 Gy). A172-SCR and A173-shAPOBEC3G were treated with or without IR (2 Gy) before being allowed to recover and grow for 10 days. Representative pictures are illustrated (A) and colony number was counted from triplicate experiments (B). (**C**) Limiting dilution sphere formation assay demonstrated that targeting APOBEC3G reduced sphere formation in GSC20 cells after IR (2 Gy). GSC20 cells were targeted with scramble or APOBEC3G shRNA, treated without or with IR (2 Gy), and diluted; 1-3 cells were plated and allowed to recover and grow for 21 days. Sphere-positive wells were counted, and the percentage of positive wells was calculated. (**D**–**E**) Immunoblot analysis showed that APOBEC3G knockdown impaired the DNA repair response after IR. A172 cells (D) and GSC20 (E) with scramble or APOBEC3G shRNA were exposed to 2 Gy IR. Cells were harvested before IR or 3 h or 12 h after IR for an immunoblot analysis with specific antibodies against phosphorylated Chk2, total Chk2, Cleaved-PARP, and APOBEC3G.

Recent reports have shown that APOBEC3G enhances lymphoma cell radioresistance by promoting DNA repair [27]. However, whether APOBEC3G influences the radioresistance of GBM cell lines is unknown. Therefore, we determined the effect of IR on A172-scramble cells and A172 APOBEC3G-shRNA cells. Cells were treated with or without IR (2 Gy) and allowed to recover and grow for 10 days. Treatment with 2 Gy of IR impaired A172 colony formation, but a significant number of cells survived (Figure 5A and 5B). Furthermore, targeting APOBEC3G sensitized A172 cells to radiation, as the combination of shAPOBEC3G and IR significantly attenuated colony formation (Figure 5A and 5B). In a limiting dilution colony formation assay, the cells were diluted and plated in each well (1–3 cells per well) of 96-well plates. Without IR, the sphere formation ability of scramble cells is similar to that of APOBEC3G knockdown cells. After 2 Gy IR, the colony formation of APOBEC3G knockdown cells was impaired by 35.1% (*P* < 0.05) compared to that of scramble cells (Figure 5C). These data suggest that targeting APOBEC3G renders mesenchymal GICs and GBM cells sensitive to IR-induced cell death.

### APOBEC3G knockdown attenuates checkpoint activation of DNA repair pathway

We next investigated the mechanism of radiosensitization in APOBEC3G knockdown cells. IR mainly causes cellular toxicity through the induction of DSBs that activate the DNA damage checkpoint signaling pathway [29, 31]. Checkpoint pathway activation initiates cell-cycle arrest with attempted DNA repair [32, 33]. Chk2 is the key checkpoint that promotes DNA repair after IR damage. As shown in Figure 5D and 5E, the expression of phosphorylated Chk2 was induced at 3 hours post 2Gy irradiation in SCR cells suggesting DNA repair is initiated. pChk2 expression was much lower in APOBEC3G knockdown cells compare with that in SCR cells at both 3 hours and 12 hours post IR, suggesting APOBEC3G depletion inhibited DNA repair. Consistently, we observed cleaved PARP, a marker for apoptosis, was induced in APOBE3G knockdown cells but not in SCR cells at 12 h post IR, demonstrating inhibition 2 Gy IR induced apoptosis in APOBE3G knocked-down cells but not in SCR cells. These data suggest that knockdown of APOBEC3G impairs activation of the DNA repair pathway and sensitizes irradiation induced- apoptosis in mesenchymal GICs and GBM cells.

## DISCUSSION

GBMs of the mesenchymal subclass have been linked with high aggressiveness and resistance to treatment, whereas patients with a proneural signature were reported to perform better in the clinic with respect to survival and treatment responses [6, 34, 35]. Up to date, many small molecule inhibitors targeting specific pathway may yield stronger specificity and thus improve killing tumor effects. Mesenchymal identity is the hallmark of GBM cell aggressiveness with tight correlation to the poor outcome of patients. In our analysis of GBM patient derived GICs, we report that in mesenchymal GICs, genes involved in TGFβ signaling pathways including APOBEC3G, is significantly up-regulated compared with non-mesenchymal GICs. Among APOBEC3 families, APOBE3G expression is the most significant unregulated target (1.8 folds and FDR = 0.0018), APOBEC3C and APOBEC3F showed moderate or slight upregulation (1.4 folds for APOBEC3C and 1.1 folds for APOBEC3F) (Supplementary Figure 1). In our studies, inhibition of APOBEC3G by specific shRNA attenuates invasion, migration and *in vitro* growth. APOBEC3G is highly expressed in clinical GBM tissues and mesenchymal GICs display a significantly higher radio resistance, with markedly elevated levels of expression of genes associated with DNA repair. Inhibition of APOBEC3G reverses the radiation-resistant phenotype of mesenchymal GICs.

A recent study demonstrated that APOBEC3G down regulates miR-29 expression and hampers miR-29 activity in repressing MMP2, which promotes hepatic metastasis of colorectal cancer [19]. Whether APOBEC3G can promote GBM cell survival and invasion is unknown. Our data show that knockdown of APOBEC3G attenuates the proliferation and invasion of GBM cell lines. We show that targeting APOBEC3G can decrease activation of the TGFβ signaling pathway, which may account for the effects of APOBEC3G on GBM. We show that targeting APOBEC3G affected TGFβ-induced smad2 activation bridging APOBEC3G and the TGFβ pathway and illustrate the functions and mechanisms of APOBEC3G in GBM. Concomitant with smad2 deactivation, we show decrease in TSP-1, MMP2, increase of TIMP-1, all are smad2 targeted genes clearly showing that targeting APOBEC3G regulates TGFβ1 signaling pathway. However, how APOBEC3G regulates the TGFβ pathway remains unknown. As a cytidine deaminase, APOBEC3G deaminates cytidine residues and causes guanosine-to-adenosine hypermutation. In addition, it suppresses translation by inhibiting the translocation of reverse transcriptase along template RNA [16] or binding to 3′UTR of target mRNA [36]. More importantly, Sharma et al. recently have discovered that APOBEC3G causes site-specific C-to-U editing of mRNAs from over 600 genes [37, 38]. Therefore, future studies focused on whether the regulation of TGFβ pathway by APOBEC3G is dependent on its ssDNA editing, elongation blockage or RNA editing function will be important to understand its mechanism and to determinate its clinical application in GBM.

Ionizing radiation is one of the most effective therapies for GBM, but radiotherapy is only palliative because of radioresistance [39]. Checkpoint pathways have cytoprotective roles that allow cells to survive after DNA repair [31]. Thus, DNA damage checkpoint responses play an important role in cellular radiosensitivity [33, 40–42]. Chk2 represents a conserved signaling component that responds to DNA damage and protects genomic integrity [43]. Activation of the Chk2 kinase in response to DNA damage is a multistep exquisite process [28, 44] and Chk2 activation can promote DNA repair after IR-induced DNA damage to render cancer cells more resistant to radiation therapy.

Several types of cancer cells, such as lymphoma and myeloma cells, display efficient repair of genomic DSBs induced by ionizing radiation and promote cell survival after IR. Interestingly, these cells express higher levels of APOBEC3G than do their paired normal cells [45]. In the current study, we identified a new function of APOBEC3G in promoting the checkpoint activation and radioresistance of GBM cell lines by regulating the key molecular checkpoint, Chk2, in cellular responses to DNA damage. Notably, depletion of APOBEC3G alone did not induce apoptosis but potentiated irradiation-induced apoptosis, suggesting that APOBEC3G plays an important role in DNA repair for irradiation-induced DNA damage and contributes to the radioresistance.

In summary, our data showed that APOBEC3G is preferentially expressed in mesenchymal gliomas and affects Smad2 activation, and enhances checkpoint activation. APOBEC3G mediated checkpoint activation through Chk2 is one of the critical regulatory mechanisms that underlies the preferential DNA damage checkpoint response and radioresistance of GICs. Thus, anti-APOBEC3G therapy may synergize with radiotherapy and other current treatments to overcome the therapeutic resistance of gliomas. APOBEC3G represents a potential molecular target for novel therapeutics that will improve the treatment outcome of glioma patients.

## MATERIALS AND METHODS

### Cell lines and reagents

Glioma initiating Cells (GICs) were established by isolating neurosphere-forming cells from surgical specimens of human GBM, as described previously [9]. These GICs were cultured as GBM neurospheres in DMEM/F12 medium containing B27 supplement (Invitrogen, Carlsbad, CA) and basic fibroblast growth factor and epidermal growth factor (20 ng/ml each). This study was approved by the institutional review board of The University of Texas MD Anderson Cancer Center (Houston, TX, USA). Both unsupervised and supervised approaches were used to classify GICs into molecular subtypes [5]. Cells were authenticated by testing short tandem repeats (STR) using the Applied Biosystems AmpFISTR Identifier kit (Foster City, CA). The last authentication testing was done in March 2014. Glioma cell lines were grown in DMEM supplemented with 10% FBS. The TGFβR1 kinase inhibitor (LY 2157299) was purchased from Selleck Chemicals (Houston, TX) and was dissolved in dimethyl sulfoxide (Sigma-Aldrich, St. Louis, MO, USA) to a concentration of 10 mmol/L. Recombinant human TGFβ1 was purchased from R&D Systems (Minneapolis, MN, USA), reconstituted in 20 μg/mL in sterile 4 mM HCl containing 1 mg/mL bovine serum albumin and stored at −20°C and further diluted to an appropriate final concentration in DMEM/F12 medium at the time of use.

### Western blot analysis

Cells were harvested in lysis solution, as previously described [46], and subjected to Western blotting analysis. Membranes were probed with the following primary antibodies: anti-phosphorylated smad2, anti-total smad2, anti-TGFβR1, anti-phosphorylated Chk2, anti-total Chk2, anti-PARP, anti-CD44, anti-Olig2 (all from Cell Signaling, Boston, MA, USA), and anti-APOBEC3G (Proteintech, Chicago, IL, USA). Anti-β-actin antibody was purchased from Sigma (St. Louis, MO, USA) and used as the loading control.

### Cell growth assay

GICs and GBM cell lines were seeded in 6-well plates (1 × 10^5^ cells/well). Cell numbers were counted every day for 4 days using a hemocytometer. The experiment was repeated three times, and the average value was reported.

### Wound healing assay

Confluent A172 and U343 cells were transfected with scramble shRNA or APOBEC3G shRNA. At 48 h after transfection, a cell scratch spatula was used to make a scratch in the cell monolayer. Pictures of the scratches were taken (× 5 magnification) using a digital camera system coupled with a microscope was taken after 18 h. The software program Image J was used to determine the migration distance (in pixels).

### Matrigel transwell migration assay

Cell migration assays were performed on polycarbonate membrane inserts (8-μm pore size; Greiner Bio-One, Inc., Longwood, FL, USA). A172 and U343 cells were washed with PBS and serum-free medium prior to being resuspended in fresh serum-free medium. 50,000 cells in 250 μl of serum-free medium were plated over the inner chamber of Matrigel-coated inserts in a 24-well tissue culture plate and 500 μl of 3% fetal bovine serum medium was placed in the outer chamber of the insert. Plates were incubated at 37°C for 24 h. The cells that had migrated through to the lower surface of the ECM layer were stained with 1% crystal violet and dissolved in 2% deoxycholic acid. Cells were quantified on the basis of the absorbance, measured at 595 nm. Each experiment was performed in triplicate.

### Knockdown of APOBEC3G by lentiviral shRNA

The lentiviral vectors pGIPZ-mediated shRNA for APOBEC3G (clone ID: V3LHS_303306) and scramble was purchased from GE Healthcare Dharmacon (Pittsburgh, PA, USA), and transfected into 293FT cells according to the manufacturer's instructions. In brief, lentiviral particles expressing targeting or control scramble shRNA were produced in HEK293FT cells with a mixed set of packing plasmids and the viruses were concentrated and titered as previously described [47]. The produced lentiviruses were concentrated using the Centricon Plus-20 centrifugal filter device (Millipore, Billerica, MA, USA). The lentiviral stock was titered and stored at −80°C. GSC20, A172, or U343 cells were infected with lentivirus for 24 hours. Cells were washed and cultured with regular complete medium for 2 days in the presence of 2.5 μg/ml puromycin. Finally, the cells were washed and analyzed for protein expression by Western blotting.

### Colony formation assay

Cells were treated with IR (2 Gy) and plated in 6-well plates (2000 cells per well), cultured in DMEM/F12 medium with 10% FBS for 10 days. Cells were stained with 1% crystal violet and dissolved in 2% deoxycholic acid. Each experiment was performed in triplicate.

### Limiting dilution sphere formation assay

Cells were treated with IR (2 Gy), and accutased to generate single-cells suspension, diluted serially to plate 1–3 cells per well in 96-well plates with 3 plates for each condition per cell line, cultured for 3 weeks DMEM/F12 medium supplemented with B27, EGF and FGF. The number of wells with neuro-sphere was counted and the percentage of positive wells was calculated. Each experiment was performed in triplicate.

### RNA extraction and real-time quantitative polymerase chain reaction

Total RNA was extracted from A172 cells using the RNeasy Mini kit (Qiagen, Valencia, CA, USA), according to the manufacturer's instructions. Real-time quantitative PCR was performed with the SuperScript III One-Step RT-PCR System and sybgreen DNA polymerase (Invitrogen, GrandIsland, NY, USA), according to the manufacturer's instructions. Primers for quantitative PCR are listed in Supplementary Table 1.

## SUPPLEMENTARY MATERIALS FIGURES AND TABLES






