# Supplementary material for: APOBEC3G acts as a therapeutic target in mesenchymal gliomas by sensitizing cells to radiation-induced cell death
Source: Oncotarget. 2017 Apr 21;8(33):54285–96. doi: 10.18632/oncotarget.17348 (PMC5589580; doi:10.18632/oncotarget.17348)
Supplement: Supplementary file 1 [file oncotarget-08-54285-s001.pdf]

# APOBEC3G acts as a therapeutic target in mesenchymal gliomas by sensitizing cells to radiation-induced cell death

## Supplementary Materials

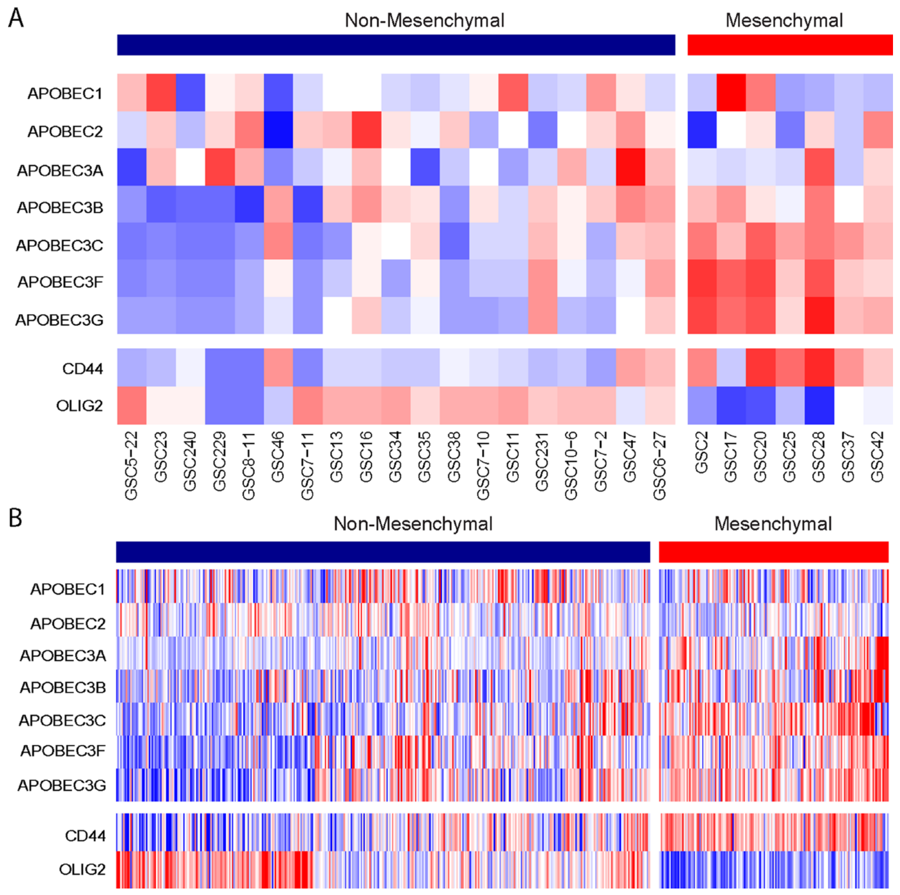


**Supplementary Figure 1:** (**A**) Gene expression analysis of APOBEC in a panel of 26 GICs are shown in the heat map. (**B**) Gene expression analysis of APOBEC in TCGA samples are shown in the heat map. CD44 is a marker of the mesenchymal subtype, whereas Olig-2 is the non-mesenchymal marker.

**Supplementary Dataset 1: mes_VS-others GICs expression.** See Supplementary_Dataset_1

**Supplementary Dataset 2: DEGinTCGA.** See Supplementary_Dataset_2

**Supplementary Table 1: Primers for Real-time quantitative PCR**

| | |
|---|---|
| GAPDH Forward | GGAGCGAGATCCCTCCAAAAT |
| GAPDH Reverse | GGCTGTTGTCATACTTCTCATGG |
| MMP2 Forward | CAGGGAATGAGTACTGGGTCTATT |
| MMP2 Reverse | ACTCCAGTTAAAGGCAGCATCTAC |
| TIMP-1 Forward | CTTCTGCAATTCCGACCTCGT |
| TIMP-1 Reverse | ACGCTGGTATAAGGTGGTCTG |
| TSP-1 Forward | CCGGCGTGAAGTGTACTAGCTA |
| TSP-1 Reverse | TGCACTTGGCGTTCTTGTT |
